# Group B Streptococcus Recto-Vaginal Colonization in Near-Term Pregnant Women, Southwest Ethiopia

**DOI:** 10.4314/ejhs.v30i5.7

**Published:** 2020-09

**Authors:** Woubishet Girma, Nadia Yimer, Tesfaye Kassa, Elias Yesuf

**Affiliations:** 1* Corresponding Author, Obstetrician and gynecologist, Department of obstetrics and gynecology, Jimma Institute of health sciences, Jimma, Ethiopia, gwubdz@yahoo.com; 2 Obstetrician and gynecologist, private hospital, Dessie, Ethiopia, nadiayimer@yahoo.com; 3 School of Medical Laboratory, Jimma University Institute of Health, Jimma, Ethiopia. ktes36@gmail.com; 4 Department of Health Economics, Management, and Policy; Jimma University Institute of Health; Jimma, Ethiopia; 5 CIH^LMU^ Center for International Health, University of Munich, Munich, Germany

**Keywords:** GBS, Recto-vaginal colonization, Near-term pregnancy, Antibiotic susceptibility, Jimma, Ethiopia

## Abstract

**Background:**

Group B Streptococcus (GBS) is recognized as an important cause of maternal and neonatal morbidity and mortality. Maternal vaginal carriage of GBS (Streptococcus agalactiae) can lead to vertical transmission to the neonate at the time of delivery. However, little is known about its prevalence, predictors and antibiotic susceptibility pattern in Jimma, Ethiopia. This study assessed the prevalence, antimicrobial susceptibility pattern and determinants of GBS recto-vaginal colonization among near-term pregnant women.

**Methods:**

A cross-sectional study was conducted from May to August 2015 at Jimma University Medical Centre in Southwest Ethiopia. Data through questionnaire and GBS isolates from vaginal and rectal swabs were collected. Antimicrobial susceptibility testing was performed.

**Results:**

The overall prevalence of GBS colonization among near term pregnant women (35–37 weeks) was 16.3% (22/135). The majority of GBS isolates were sensitive to Ampicillin and Penicillin G with 95.5% and 90.1%, respectively. Erythromycin and clindamycin were resisted by 50% and 40.9% of the isolates, respectively, whereas gentamicin was resisted by all isolates. GBS colonization was significantly associated with a history of preterm delivery (PTD) (AOR: 6.3, 95% CI: 1.42, 28.3) and history of urinary tract infection (UTI) during current pregnancy (AOR: 6.4, 95% CI, 1.95, 21.1).

**Conclusion:**

Our study indicated that one among six near-term pregnant women had recto-vaginal GBS colonization. In places where universal screening is not feasible, selective screening for factors particularly history of PTD and UTI during current pregnancy may be a reasonable option. Antibiotic susceptibility testing should be performed while using Erythromycin, Clindamycin or Gentamicin.

## Introduction

Group B streptococcus (GBS) is a member of gram-positive streptococci. GBS, also known as *Streptococcus agalactiae*, is found in 15 to 40% of healthy women's colon and vagina (1–3). Maternal colonization of GBS is associated with an increased risk of urinary tract infection (UTI), endometritis, chorioamnionitis, sepsis and meningitis (3–8). Moreover, pregnant mothers may develop a premature rupture of membranes, stillbirth and low birth weight babies (9,10). Obvious GBS diseases in the first week of delivery are referred to as Early-Onset Disease (EOD), whereas Late-Onset Diseases (LOD) associated with GBS happens between one week to three months after delivery (1–3).

The prevalence of vaginal colonization by GBS among pregnant women varies across the world, it has been estimated at 9.1% to 26.7% in Iran (11), and 8% – 15% in Europe (12). The prevalence of GBS in parts of Africa such as in Egypt, Malawi, Nigeria, and Tanzania ranges from 11.3% to 23% (13–16). Prevalence of GBS recto-vaginal colonization among pregnant women has also been studied at different parts of Ethiopia including from Addis Ababa, Gondar, Adigrat, Mekele, Hawasa, and Jimma that ranges from 7.2% to 20.9% (17–22).

Universal screening of pregnant women at 35–37 weeks of gestation for GBS by rectovaginal culture is recommended so that appropriate intrapartum antibiotic prophylaxis (IAP) can be administered for positive cases (1). In the United States, since the early 1990s, medical interventions have resulted in an 80% decline in the incidence of early-onset neonatal sepsis due to GBS from 1.7 to less than 0.4 cases per 1000 live births in recent years (7). There are concerns about the emergence of drug resistance in parts of the world where IAP is practiced (1,23–26). In this study's location, updated information on the trends of drug resistance on commonly used drugs is needed for practicing professionals.

Socio-demographic characteristics like greater age, increased parity, low socio-economic status, obesity level, place of residence (urban vs. rural) have been associated with increased risk of maternal colonization of GBS (9,27). In Ethiopia, like other resource-limited areas, there are scanty studies conducted to assess the prevalence of recto-vaginal GBS colonization. Furthermore, screening of pregnant women for GBS is not at all routine practice. Therefore, this study assessed the prevalence of rectovaginal colonization, antibiotic susceptibility patterns and determinants of GBS isolated from near term pregnant women at Jimma University Medical Center (JUMC).

## Materials and Methods

**Study setting**: The study was conducted at JUMC located in the town of Jimma, 355 km Southwest of Addis Ababa. JUMC is the only teaching and referral hospital in the southwestern part of Ethiopia with a catchment of 15 million people. The center has bed occupancy of 650, of which 52 beds belong to the obstetric ward. The hospital gives 24-hour service of attending normal and abnormal labor and basic and specialized antenatal care (ANC) follow-ups.

A cross-sectional study was conducted from May 1^st^ to August 31^st^, 2015.

The study population consisted of nearterm pregnant women with gestational ages of 35 to 37 weeks, who attended ANC clinic at JUMC during the study period.

**Sample size**: A sample size was determined using a single proportion formula with a 95% confidence level (Z of 1.96), and a standard error of 0.05. The minimum sample size (n) was calculated by taking the carriage rate of *S. agalactiae* (GBS) in pregnant women with a 9% carriage rate from a previous study done in northern Ethiopia (18). Ten percent of the total calculated sample size (n=126) was added for the non-response rate, which made the sample size 139. Among the 139 pregnant women, four refused to participate in the study. Thus, a total of one hundred thirty-five (135) pregnant women were included in the analysis yielding a 97% response rate.

**Data collection**: Data were collected from near-term pregnant women through a non-probability consecutive sampling technique using a checklist within the study period by two trained midwives. Demographic characteristics and clinical outcomes of interest were collected using a well-structured questionnaire through interview technique using tools from previous studies (6,7&^17^–^20^).

For laboratory work, specimens from the lower third of the vagina and rectum were collected using sterile cotton-tipped swabs in separate sterile tubes of Ames transport medium by a trained midwife and a gynecologist. It was immediately transported to the microbiology laboratory of JUMC within one hour of collection. The specimens were inoculated directly into 5% sheep blood agar (Oxoid Limited, Hampshire, UK) plates supplemented with 8 µg/ml gentamicin and 15 µg/ml nalidixic acid (Biomerieux, France) to prevent the growth of contaminants. The plates were incubated at 37°C aerobically for 24 hours. Cultures were examined for beta-hemolytic streptococci colony, and all negative plates were re-incubated for an additional 24 hours. Suspicious GBS colonies were identified by colony morphology, gram stain and biochemical tests including catalase, sodium hippurate hydrolysis, and Christie, Atkins, Munch-Petersen (CAMP) factor positivity (4,28). Antimicrobial susceptibility of all GBS isolates was determined for commonly used antibiotics including Penicillin G (10µg), Ampicillin (10µg), Erythromycin (15µg), Clindamycin (2µg) and Gentamicin (30µg) by using Kirby-Bauer disk diffusion method on Mueller-Hinton agar supplemented with 5% defibrinated sheep blood. The susceptibility results were interpreted according to the recommendations of the Clinical and Laboratory Standards Institute (CLSI) guidelines for beta hemolytic streptococci in 2012(29).

Women who were found colonized by GBS were notified by phone and told to visit the ANC clinic. Memos were produced to the attending physician indicating that intrapartum antibiotics should be given at the onset of true labor.

**Statistical analysis**: Collected data were used to determine associations of selected variables like socio-demographic characteristics and obstetric and medical histories with positive recto-vaginal GBS colonization using SPSS for windows version 21.0. Bivariate analysis was carried out for identified categorical variables and a p-value of ≤ 0.25 was used as a cut-off point to select candidate variables for the final multiple logistic regression model. Independent predictors including history of contraception, early-onset neonatal sepsis, premature rupture of membranes, preterm delivery, UTI and other variables were determined using adjusted odds ratio with 95% confidence interval in multiple regression analysis at p-value < 0.05.

## Results

**Socio-demographic characteristics**: The age of the study participants ranged from 16 to 38 years with a mean of 24.9 (± 4.5) years. The majority (62.2%) of the study participants were urban residents, married (95%) and Muslim (68%) by religion. Thirty-one (23%) of them had educational level of beyond secondary school (Table 1).

**Table 1:** Socio-demographic characteristics of near-term pregnant women involved in the study at JUMC, Jimma, Ethiopia from May to August, 2015 (n=135)

Sociodemographic factor	Frequency	Percentage
**Age group**		
Mean age	24.9 (± 4.5) years	
15–19 years	8	5.9%
20–24 years	57	42.2%
25–29 years	47	34.8%
30–34 years	16	11.9%
≥35 years	7	5.2%
**Residential location**		
Urban	84	62.2%
Rural	51	37.8%
**Religion**		
Muslim	91	67.5%
Orthodox	31	22.9%
Protestants	13	9.6%
**Educational status**		
No formal education	28	20.7%
Primary / Secondary	76	56.3%
Post-secondary	31	23%
**Monthly income**		
Mean income	2050.6(± 1145.6) Birr	
<1800 Birr	39	28.8%
1800–2400 Birr	52	38.5%
>2400 Birr	44	32.6%
**Marital status**		
Married	128	94.8%
Single	7	5.2%

**Obstetric characteristics**: More than half of the women (55.6%) were multigravida and 40.7% of them were in the 37^th^ week of their pregnancy. Fifteen (11.1%) of the participants had a history of preterm birth, 5.2% had premature rupture of membranes (PROM), and only 3% had neonates with previous early-onset neonatal sepsis (EONS). Contraception use among the study subjects was 65.2% (Table 2).

**Table 2:** Obstetric and medical characteristics of near-term pregnant women involved in the study at JUMC, Jimma, Ethiopia from May to August, 2015 (n=135)

Obstetric variables	Frequency	Percentage
**Gravidity**		
1	60	44.4%
2–4	63	46.7%
≥5	12	8.9%
**Gestational age**		
35 weeks	*51*	37.8%
36 weeks	29	21.5%
37 weeks	*55*	40.7%
**Previous ANC visit**	131	97%
**History of PTD**	13	17.3%
**History of PROM**	7	5.2%
**History of new-born with EONS**	4	3%
**Contraception use**	88	65.2%
**UTI during current pregnancy**	28	21%
**STI during current pregnancy**	3	2%
**HIV infection**	4	3%

**Hypertension**	8	6%

**JUMC:** Jimma University Medical Center; **ANC:** Antenatal care; **PTD:** Preterm delivery; **PROM:** Premature rupture of membranes; **EONS:** Early onset neonatal sepsis; **UTI:** Urinary tract infection; **STI:** sexually transmitted infections; **HIV:** Human immune deficiency virus

**Medical characteristics**: In our study, 21% of the study participants had history of UTI during the current pregnancy. Only three participants had a history of sexually transmitted infections (STI). Testing for human immunodeficiency virus (HIV) was done for all participants, and four women were found to be seropositive. Eight women had a history of hypertension (HTN) (Table 2).

**Figure 1:**
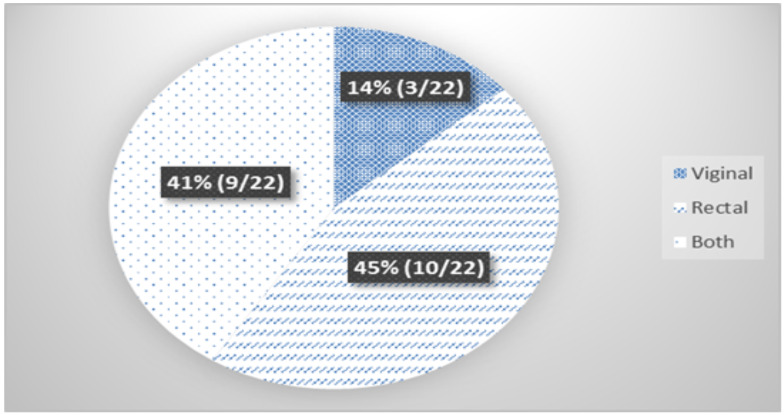
Site of recovery of GBS colonization among pregnant women attending ANC JUMC, Jimma, Ethiopia from May to August, 2015 (n=22)

**Prevalence of Group B *Streptococci***: The prevalence of GBS colonization among the study subjects was 16.3% (22/135). Of all the GBS isolates identified, nearly half [45% (10/22)] of them were recovered from the rectum only. In 9/22(41%) of the study subjects, GBS isolates were found from both rectum and vagina (Figure 1).

**Antimicrobial susceptibility testing**: Twenty-one (95.5%) and 20 (90.9%) of the 22 GBS isolates were susceptible to Ampicillin and Penicillin G, respectively. Only two isolates (9%) were found to be susceptible to Clindamycin. Furthermore, intermediate susceptibility was also detected against Erythromycin in one isolate (4.5%), and Clindamycin in three isolates (13.6%). However, eleven (50%) and nine (40.9%) isolates were resistant to Erythromycin and Clindamycin, respectively. All of the isolates showed resistance to Gentamicin (100%) (Table 3).

**Table 3:** Antimicrobial susceptibility pattern of GBS isolated from near term pregnant women at JUMC, Jimma, Ethiopia from May to August, 2015 (n=135)

Antibiotics	Disc potency(µg)	Susceptible	Intermediate	Resistance
Ampicillin	10	21(95.5%)	-	1(4.5%)
Penicillin G	10 U	20(90.9%)	-	2(9.1%)
Erythromycin	15	10(45.5%)	3(13.6%)	11(50%)
Clindamycin	2	10(45.5%)	1(4.5%)	9(40.9%)
Gentamicin	30	-	-	22(100%)

**GBS Colonization or its absence among near term pregnant women**: In univariate analysis, completing primary or secondary education, history of preterm delivery (PTD) and history of UTI during current pregnancy were significantly associated with recto-vaginal GBS colonization, while the other socio-demographic, obstetric and medical characteristics were not significantly associated with recto-vaginal GBS colonization (Table 4).

**Table 4:** Determinants of GBS colonization among near term pregnant women at JUMC, Jimma, Ethiopia from May to August, 2015 (n=135)

Variables	GBS		COR:95%,CI	AOR:95%,CI

	Positive (N=22)	Negative (N=113)		
**Age group**				
≤24 years	13(20%)	52(80%)	1.14(0.34–3.85)	2.5(0.23–27.3)
25–29 years	5(10.6%)	42(89.4%)	0.54(0.13 – 2.18)	0.15(0.01–2.65)
≥30 years	4(17.4%)	19(82.6%)	1*	1*
**Residential location**				
Urban	14(16.7%)	70(83.3%)	1.34(0.52 – 3.46)	2.4(0.30–19.6)
Rural	8(15.6%)	43(84.4%)	1*	1*
**Educational status**				
No formal education	6(21.4%)	22(78.6%)	0.67(0.21 – 2.15)	1.471(0.25–8.78)
Primary or secondary education	7(9.2%)	69(90.8%)	0.25(0.08 – 0.73)	0.53(0.11–2.52)
Post-secondary education	9(29%)	22(71%)	1*	1*
**Marital status**				
Married	20(15.6%)	108(84.4%)	0.47(0.09–2.27)	0.65(0.02–17.8)
Single	2(28.6%)	5(71.4%)	1*	1*
**Gravidity**				
1	10(16.7%)	50(82.3%)	1.19(0.35–4.0)	4.14(0.33–52.3)
2–4	10(15.8%)	53(84.2%)	0.57(0.14–2.29)	1.7(0.18–16.2)
≥ 5	2(16.6%)	10(83.4%)	1*	1*
**History of** **contraception**				
Yes	16(18.2%)	72(81.8%)	1.52(0.52–4.15)	1.11(0.28–4.39)
No	6(12.8%)	41(87.2%)	1*	1*
**History of PTD**				
Yes	6(40%)	9(60%)	4.33(1.35–13.81)	6.35(1.42–28.35)†
No	16(13.3%)	04(86.7%)	1*	1*
**History of PROM**				
Yes	2(28.5%)	5(71.5%)	2.0(1.41–9.82)	3.32(0.41–26.9)
No	20(15.6%)	108(84.4%)	1*	1*
**History of UTI during** **current pregnancy**				
Yes	11(39.3%)	17(60.7%)	5.6(2.11–15.1)	6.4(1.95–21.1)†
No	11(10.2%)	96(89.8%)	1*	1*

N.B. 1* - reference

† statistically significant at P-Value < 0.05

COR- Crude Odds Ratio, AOR- Adjusted Odds Ratio

**Predictors of recto-vaginal GBS colonization**: Results from the multiple logistic regression analysis are presented in Table 4. Adjusting for socio-demographics, obstetric and medical characteristics, history of PTD and UTI during current pregnancy were significantly associated with GBS colonization. Women with previous preterm delivery were 6.3 times more likely to be colonized compared to those women with no history (AOR: 6.3, 95% CI: 1.42, 28.3). Women with history of UTI during current pregnancy were 6.4 times more likely to be colonized by GBS (AOR: 6.4, 95% CI: 1.95, 21.1) compared to women with no such history. Completing primary or secondary school and being employee were not associated with GBS.

## Discussion

Our study examined the prevalence as well as predictors of colonization, and antibiotic susceptibility of GBS isolates among near term pregnant women in Southwest Ethiopia. Our study showed a 16.3% prevalence of GBS rectovaginal colonization. This finding is in the range of the USA prevalence reports of 15–40% (3) and other studies done in parts of Africa: Egypt: 17.89% (15), Malawi: 16.5% (14), Nigeria: 11.3% (13), Tanzania: 23% (16), and Democratic Republic of Congo (DRC): 20% (29). The result is also comparable to most of the studies done in Ethiopia with a prevalence range of 11.3%–20.9% (19–22). However, a lower prevalence of recto-vaginal colonization was reported in south India: 2.3% (30), Mozambique: 1.8% (31), and two Ethiopian studies done earlier with 7.2% in Addis Ababa (17) and 9% in Gondar (18). The variation in prevalence at different places could be due to differences in sample size and study period, as our study was a cross-sectional study during a shorter period. Additionally, there is variation in the GBS colonization rate when sampled from rectal and/or vaginal sites of the body, and identification of GBS from clinical specimens varies from one study to another.

Identification of risk factors associated with GBS colonization has a paramount effect in reducing maternal and neonatal complications in resource-limited countries like Ethiopia, where routine screening is not the practice (32). In this study, risk factors associated with GBS colonization were history of preterm birth and UTI during the current pregnancy. History of both PTD and UTI during the current pregnancies were associated with six-fold higher odds of GBS colonization. Similar findings have been reported in a study done in the DRC (29). In women with history of PTD or UTI, it is several times as likely to have GBS colonization compared to those who don't have either history. In this instance, GBS colonization is one of the agents associated with PTD or UTI.

In our study, the majority of the isolates were susceptible to Ampicillin (95.5%) and Penicillin G (90.9%), which is different from most studies conducted previously in which all isolates were susceptible to these two drugs (16, 20–22,33). The United States of America Centers for Disease Control and prevention (CDC) recommends that penicillin remains the agent of choice for IAP, with Ampicillin as an acceptable alternative to prevent GBS colonization and infection (7). This study claims that, considering the observed low rates of GBS resistance to penicillins, it is mandatory to extend the study on the GBS colonization rate among near term pregnant women with a larger number of study participants over a longer period of time. Besides, since there is no treatment guideline in this study location, it would be logical to consider GBS culture and susceptibility testing routinely to select for effective antimicrobial agents.

According to the USA CDC guidelines, penicillin-allergic women at high risk for anaphylaxis ought to receive Clindamycin if their GBS isolate is susceptible to Clindamycin and Erythromycin (7). In this study, half of the isolates were resistant to Erythromycin. This is consistent with reports from other studies done in Nigeria, 35.3% (13), and Palestine, 43% (33). The findings of this study also indicated that 40.9% of the isolates were resistant to Clindamycin. Gentamicin, on the other hand, showed 100% resistance to GBS isolates in our study that has been used in combination with penicillin for severe GBS associated infections (34). These increased rates of GBS resistance to Erythromycin, Clindamycin, and Gentamicin highlight the importance of mandatory antimicrobial susceptibility testing in all GBS isolated from pregnant women.

The strengths of this study are the more valid method used to identify GBS colonization, which is culture, and the prospective nature of data collection. However, this study is limited given the fact that it is a cross-sectional study to establish temporality between risk factors and GBS colonization. Moreover, since the participants were approached in a health facility, the findings may not be generalizable to the target population.

In conclusion, the prevalence of GBS rectovaginal colonization among near-term pregnant women is comparable to most of the studies done in Ethiopia and most parts of the world. In resource-limited areas, where universal screening is not feasible, selective screening for the identified predictors may be a reasonable option. Further study to identify risk factors for rectovaginal colonization at community level is recommended. Antibiotic susceptibility of the bacterium to Ampicillin and Penicillin G was high. The increased resistance rates to both Erythromycin and Clindamycin emphasize that antibiotic susceptibility tests should be done in women with allergy to penicillin. GBS is resistant to Gentamycin; reconsideration must occur before using this drug for GBS related infections until patient specific culture and antibiotic profile is known.
